# Expression, purification, and characterization of diacylated Lipo-YcjN from *Escherichia coli*

**DOI:** 10.1016/j.jbc.2024.107853

**Published:** 2024-10-01

**Authors:** Matthew A. Treviño, Kofi A. Amankwah, Daniel Fernandez, Scott A. Weston, Claire J. Stewart, Jaime Morales Gallardo, Mona Shahgholi, Naima G. Sharaf

**Affiliations:** 1 Department of Biology, Stanford University, Stanford, California, USA; 2 Macromolecular Structure Knowledge Center (MSKC) at Sarafan ChEM-H, Stanford University, Stanford, California, USA; 3 Sarafan ChEM-H Institute, Stanford University, Stanford, California, USA; 4 Division of Chemistry and Chemical Engineering, California Institute of Technology, Pasadena California, USA

**Keywords:** substrate binding proteins, X-ray crystallography, *Escherichia coli*, bacterial lipoproteins, recombinant protein expression

## Abstract

YcjN is a putative substrate binding protein expressed from a cluster of genes involved in carbohydrate import and metabolism in *Escherichia coli*. Here, we determine the crystal structure of YcjN to a resolution of 1.95 Å, revealing that its three-dimensional structure is similar to substrate binding proteins in subcluster D-I, which includes the well-characterized maltose binding protein. Furthermore, we found that recombinant overexpression of YcjN results in the formation of a lipidated form of YcjN that is posttranslationally diacylated at cysteine 21. Comparisons of size-exclusion chromatography profiles and dynamic light scattering measurements of lipidated and nonlipidated YcjN proteins suggest that lipidated YcjN aggregates in solution *via* its lipid moiety. Additionally, bioinformatic analysis indicates that YcjN-like proteins may exist in both Bacteria and Archaea, potentially in both lipidated and nonlipidated forms. Together, our results provide a better understanding of the aggregation properties of recombinantly expressed bacterial lipoproteins in solution and establish a foundation for future studies that aim to elucidate the role of these proteins in bacterial physiology.

ATP-binding cassette (ABC) transporter systems are present in all organisms and mediate the transport of molecules across cellular membranes (1). These systems share a conserved architecture consisting of transmembrane domains (TMDs) that act as a channel for ligand transport and nucleotide binding domains (NBDs) that hydrolyze ATP. Some ABC transporters also associate with substrate binding proteins (SBPs) that bind molecules and deliver them to their cognate ABC transporter (2). To create a functional complex, SBPs and ABC transporters must be delivered to their appropriate subcellular locations. In diderm bacteria, which contain an inner membrane (IM) and an outer membrane, SBPs are secreted through the IM into the periplasm and ABC transporters are embedded within the IM. The appropriate assembly of an ABC transporter ensures that TMDs and NBDs can access periplasmic SBPs and cytoplasmic ATP, respectively (Fig. 1*A*).

**Figure 1:**
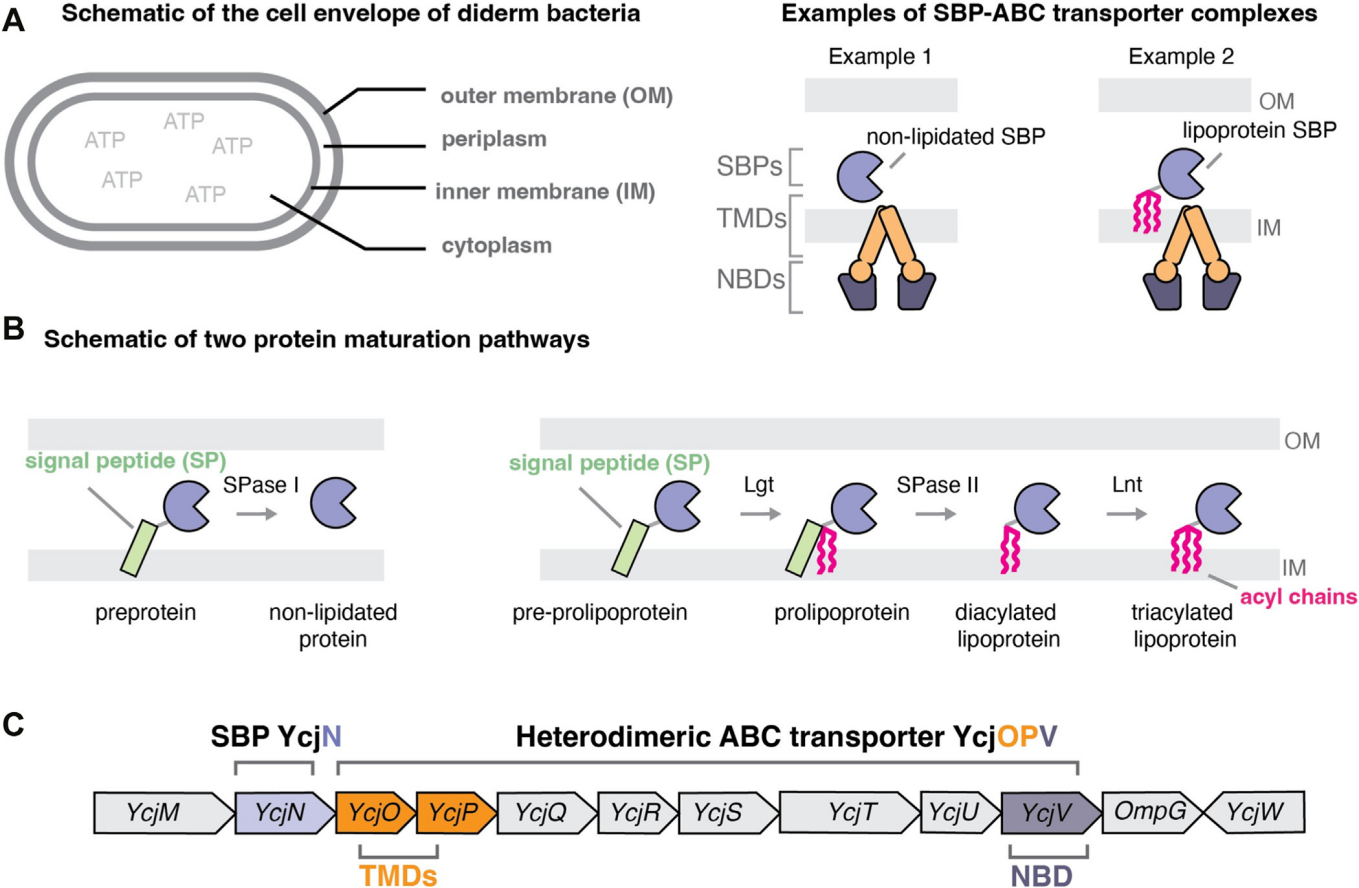
**Subcellular localization and posttranslational modification pathways of SBPs**. *A*, diagram of the cell envelope (*left panel*) and organization of ABC transporter systems in complex with a soluble SBP and a lipoprotein SBP (*right panel*), respectively. *B*, nonlipidated protein maturation pathway (*left panel*) and lipoprotein maturation pathway (*right panel*). *C*, representation of the *ycj* gene cluster in *E. coli*. The protein encoding genes for the SBP, TMDs, and NBD are colored in *light blue*, *orange*, and *dark blue*, respectively. ABC, ATP-binding cassette; NBD, nucleotide binding domain; SBP, substrate binding protein; TMD, transmembrane domain.

Since SBPs are produced in the cytoplasm, they must first traverse the IM as immature preproteins. While attached to the IM, these proteins can undergo a series of posttranslational modifications. Studies on protein maturation have revealed that this process depends on distinct protein segments, specifically the N-terminal signal peptide (SP) (Fig. 1*B*, green rectangles). For example, preproteins destined to mature into untethered secreted soluble proteins (referred to here as nonlipidated proteins) contain an SP recognized by type I signal peptidase (SPase I) (Fig. 1*B*, left panel). This enzyme cleaves the C terminal region of the SP from the IM-tethered preprotein, releasing the mature protein into the periplasm (3). Alternatively, preproteins that mature into lipoproteins contain an SP recognized by phosphatidylglycerol:prolipoprotein diacylglyceryl transferase (Lgt) (Fig. 1*B*, right panel). This enzyme adds a diacylglyceryl group from phospholipids to the sulfhydryl group of the invariant cysteine within the C terminal region of the SP. Type II signal peptidase (SPase II) then cleaves the SP so that the S-diacylglyceryl-cysteine becomes the new N terminus. A third enzyme, apolipoprotein N-acyltransferase (Lnt), then acylates the amino group of cysteine to produce a triacylated N-acyl S-diacylglyceryl-lipoprotein (Fig. 1*B*, right panel) (4). To date, most SBPs characterized in diderm bacteria mature into nonlipidated SBPs. However, some SBPs have been experimentally determined to be lipoproteins (5, 6, 7).

SBPs associate with ABC transporters to scavenge nutrients from their surroundings, such as sugars, trace metals, and amino acids (8, 9, 10). Despite their low sequence similarity, SBPs have a highly conserved three-dimensional fold that consists of two *α*/*β* domains and a hinge region with one to three segments. Ligand binding causes SBP conformational rearrangements that result in the formation of a ligand binding pocket at the interface between these domains (11). In this pocket, the SBP and the ligand form an extensive network of hydrophobic interactions and hydrogen-bond contacts, which influence ligand binding specificity (8, 9, 10). A previous study grouped SBPs into seven clusters (A-G) based on structural similarity (11). While there is some correlation between the cluster and the ligand specificity, SBPs within the same cluster can bind to a wide range of ligands (11). This observation suggests that SBP ligand specificity cannot be determined by structural comparison alone and should be verified experimentally.

YcjN is a poorly characterized putative SBP in *Escherichia coli*, whose protein-encoding gene is located within a cluster of 12 genes (12). In this cluster, three genes are predicted to encode protein components of an ABC transporter, including two TMDs (YcjO and YcjP) and an NBD (YcjV). Additionally, this operon also encodes two sugar dehydrogenases (YcjS and YcjQ), two phosphorylases (YcjT and YcjM), a *β*-phosphoglycomutase (YcjU), an epimerase/isomerase (YcjR), a predicted LacI-type repressor (YcjW), and an outer-membrane porin believed to be involved in the nonspecific import of oligosaccharides (OmpG) (Fig. 1*C*). Furthermore, previous studies using radioactive palmitate labeling have shown that YcjN is a lipoprotein (6). However, YcjN’s site of lipidation, molecular structure, and ligand binding specificity remain unknown.

Here, we used various biophysical tools to characterize *E. coli* YcjN. Specifically, using LC-MS of recombinantly overexpressed YcjN, we show that it is heterogeneously diacylated at cysteine 21. A comparison to its nonlipidated protein form (ΔYcjN) using dynamic light scattering (DLS) and negative stain transmission electron microscopy (TEM) also reveals that Lipo-YcjN aggregates *via* its lipid moiety in solution. We also report the first crystal structure of non-lipidated YcjN form (ΔYcjN) to a resolution of 1.95 Å. A structural comparison of YcjN to previously characterized SBPs reveals that YcjN can be classified into subcluster D-I and is structurally similar to the well-characterized maltose binding protein (MBP). Next, we investigated the taxonomic distribution of YcjN-like proteins using bioinformatic tools. Our analyses suggest that YcjN-like proteins are present in both Bacteria and Archaea. Together, our work provides a more detailed understanding of the aggregation properties of Lipo-YcjN in solution and establishes a foundation for future studies aimed at elucidating the role of YcjN in bacterial physiology.

## Results

### Recombinantly overexpressed *E. coli* YcjN is a lipoprotein heterogeneously lipidated at cysteine 21

**Figure 2:**
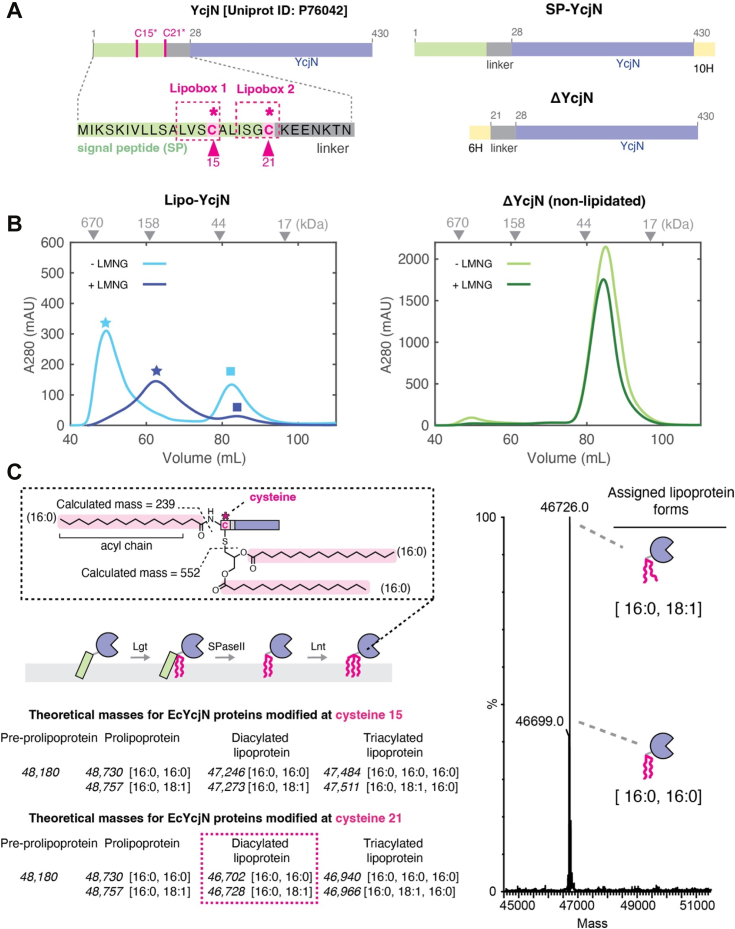
**Purification and mass spectrometry analysis of YcjN proteins**. *A*, diagram of the amino acid sequences of YcjN proteins. Full-length YcjN contains two distinct lipoboxes (*pink dashed boxes*) within its SP (*green rectangle*). Each lipobox contains a cysteine residue (15 and 21, *pink triangles*). *B*, SEC elution profiles of Lipo-YcjN purified in the presence and absence of LMNG are shown in *dark* and *light blue*, respectively. SEC elution profiles of ΔYcjN purified in the presence and absence of LMNG are shown in *dark* and *light green*, respectively. Peak elution volumes of gel filtration standards are indicated by *gray triangles*. *C*, the theoretical masses of YcjN proteins modified at cysteine 15 (*top*) or cysteine 21 (*bottom*) are calculated, assuming that triacylation occurs *via* the canonical triacylation pathway due to the sequential action of three enzymes (Lgt, SPase II, and Lnt). *Gray dashed box* insert contains illustration of acyl chains attached to a C-terminal cysteine and the average calculated acyl chain masses of a lipoprotein with acyl chain composition [16:0, 16:0, 16:0]. Mass spectrum of the peak fraction (elution volume of 62 ml, *dark blue star*) of Lipo-YcjN purified in the presence of LMNG. The molecular masses of the major species correspond within 3 Da of the predicted masses of two diacylated YcjN proteins modified at residue 21, one protein with an acyl chain composition [16:0,16:0] and another with [16:0,18:1] (*right panel*). Assigned lipoprotein forms are shown as illustrations on the *right*. Lgt, phosphatidylglycerol: prolipoprotein diacylglyceryl transferase; LMNG, 2,2-didecylpropane-1,3-bis-β-D-maltopyranoside; SEC, size-exclusion chromatography; SP, signal peptide; SPase II, type II signal peptidase.

The SPs of bacterial lipoproteins often contain a consensus sequence [LVI][ASTVI][GAS]-[C] named the lipobox motif that is located within the first 40 N-terminal residues (13). A visual inspection of YcjN’s SP revealed two lipobox motifs, [L_12_VSC_15_] and [I_17_SGC_21_], each containing a cysteine residue with the potential to be modified with lipids (cysteine 15 and 21) (Fig. 2*A*, pink triangles). To determine whether cysteine 15 or cysteine 21 is lipidated, we recombinantly overexpressed YcjN in *E. coli* with its native N-terminal SP and a C-terminal decahistidine tag to aid in protein purification (SP-YcjN) (Fig. 2*A*). The full-length SP-YcjN protein was then purified using affinity followed by size-exclusion chromatography (SEC) in the presence of the detergent 2,2-didecylpropane-1,3-bis-β-D-maltopyranoside (LMNG) (Fig. 2*B*, dark blue trace). The SEC elution profile displayed major and minor peaks with elution volumes of 62 ml and 84 ml, respectively (Fig. 2*B*, dark blue star and square, respectively). SDS-PAGE analysis of peak fractions revealed prominent bands close to the theoretical molecular weight of SP-YcjN (48,180 Da) (Fig. S1*A*). These data suggest that we successfully expressed and purified recombinant SP-YcjN. Furthermore, SEC and SDS-PAGE analyses reveal that there are two species of SP-YcjN with similar molecular weights but with different elution volumes.

To determine whether cysteine 15 or cysteine 21 is lipidated, we used LC-MS for intact protein analysis of the major peak fraction of SP-YcjN purified in the presence of LMNG (Fig. 2*B*, dark blue star marker). This method was selected because of the distinct theoretical mass differences between YcjN lipoprotein forms. For example, the mass difference between the triacylated YcjN lipoproteins at cysteine 15 and cysteine 21 is approximately 545 Da (Fig. 2*C*, left panel). Mass spectrometry analysis showed deconvoluted mass values of 46,699 Da and 46,726 Da (Fig. 2*C*), aligning closely with the theoretical masses of YcjN lipoproteins modified at cysteine 21 with diacyl chain compositions of [16:0, 16:0] (46,702 Da) and [16:0, 18:1] (46,728 Da), respectively. The numbers in square brackets indicate the total number of carbons and double bonds in each acyl chain, respectively. The observed mass values do not match the theoretical masses of YcjN proteins lipidated at cysteine 15, which would be approximately 47,500 Da. These findings suggest that recombinantly overexpressed lipidated YcjN is predominantly heterogeneously diacylated at cysteine 21 with acyl chain compositions of [16:0, 16:0] and [16:0, 18:1] (referred to as Lipo-YcjN).

### Lipo-YcjN can be purified with and without LMNG

In our previous study, we used detergents for the purification of *Neisseria meningitidis* lipoprotein MetQ (5). We reasoned that detergents would facilitate the extraction of this lipoprotein from cellular membranes. However, it was unknown whether the detergent is essential for the purification of all bacterial lipoproteins. To determine whether diacylated Lipo-YcjN can be purified in the absence of detergent, we repeated the purification of Lipo-YcjN in detergent-free conditions. A representative SEC profile of Lipo-YcjN purified in the absence of detergent is shown in Figure 2*B* (light blue trace). This profile exhibited a major peak with an elution volume of 49 ml (Fig. 2*B*, light blue star) and a minor peak with an elution volume of 82 ml (Fig. 2*B*, light blue square). An SDS-PAGE analysis of the peak fractions revealed prominent bands close to the theoretical molecular weight of Lipo-YcjN (46,702 Da) (Fig. S1*B*). To specifically identify Lipo-YcjN forms, we analyzed both the major and minor SEC peak fractions using LC-MS. Mass spectrometry analysis of the major peak with an elution volume of 49 ml (Fig. 2*B*, light blue star) revealed deconvoluted mass values of 46,699 Da and 46,727 Da (Fig. S1*C*). These values correspond well with theoretical mass values of Lipo-YcjN forms with diacyl chain compositions of [16:0,16:0] (46,702 Da) and [16:0, 18:1] (46,728 Da). These mass values are similar to those obtained with the previous measurements of Lipo-YcjN purified in the presence of LMNG (Fig. 2*C*, right panel), revealing that similar Lipo-YcjN protein forms can be purified in the presence and absence of detergent. We also performed mass spectrometry analysis of the minor peak fraction (Fig. 2*B*, light blue square). This analysis revealed the presence of various low intensity mass peaks between (46,000 and 47,500) with two deconvoluted masses of high intensity of 46,521 Da and 46,993 Da (Fig. S1*D*). These mass values do not align well with YcjN forms from the canonical lipoprotein maturation pathway (Fig. 2*C*, left panel). However, it is possible that masses are YcjN proteins that have undergone degradation or noncanonical N-terminal cleavage of the SP. For example, these values correspond well to an YcjN protein with an N-terminal cleavage between alanine 16 and leucine 17 (theoretical mass: 46,521 Da) and another YcjN protein cleaved between alanine 11 and leucine 12 (theoretical mass: 46,995 Da). However, due to the presence of various low intensity peaks around the same mass range we are not confident in these assignments. Together, these data show that detergent is not necessary for the purification of recombinantly overexpressed diacylated Lipo-YcjN using chromatography techniques, and that heterogeneously diacylated Lipo-YcjN with acyl chain composition of [16:0, 18:1] and [16:0, 16:0] can be successfully obtained both with and without detergent.

### Lipo-YcjN aggregates *via* its lipid moiety

The SEC profile of Lipo-YcjN purified in the absence of LMNG reveals that its major peak elution volume is lower than anticipated (Fig. 2*B*, light blue star). Specifically, we expect proteins with masses similar to monomeric Lipo-YcjN (46,702 Da, theoretical molecular weight of YcjN heterogeneously lipidated at residue 21) to elute at approximately 85 ml, as determined by the peak elution volumes of gel filtration standards (Fig. 2*B*, gray triangles). However, Lipo-YcjN elutes at 49 ml, an elution volume similar to that of a gel filtration protein standard with a molecular weight close to 670,000 Da (Fig. 2*B*, light blue star). These results suggest that Lipo-YcjN aggregates in solution.

To determine whether Lipo-YcjN aggregation is mediated primarily by its globular domain or lipid moiety, we designed a construct with an SP deletion (ΔYcjN, residues 1–21 deleted) (Fig. 2*A*). In this experiment, if aggregation is driven primarily by the protein’s globular domain, we would anticipate that both ΔYcjN and Lipo-YcjN would elute at similar volumes. In other words, aggregation is not dependent on the presence or absence of the lipid moiety. However, if Lipo-YcjN aggregation is primarily driven by the lipid moiety, we would expect ΔYcjN and Lipo-YcjN to elute at different volumes, indicative of lipid-dependent aggregation. The elution profiles of recombinantly expressed ΔYcjN in the presence and absence of LMNG are shown in Figure 2*B*, right panel. In the presence of LMNG, ΔYcjN elutes at 84 ml (Fig. 2*B*, dark green trace), while in its absence, ΔYcjN elutes at 85 ml (Fig. 2*B*, light green trace). These peak elution volumes correspond well with the expected elution volume for a protein with a theoretical molecular weight of monomeric ΔYcjN (45,639 Da). Together, these results suggest that Lipo-YcjN, but not ΔYcjN, aggregates in solution. To confirm the aggregation of the Lipo-YcjN protein in solution, we performed DLS measurements on purified YcjN proteins. We also used MBP as a control. The DLS measurements revealed that the hydrodynamic radius (Rh) of MBP was 3.0 *±* 0.1 nm (Fig. S2*A*), while the Rh of ΔYcjN was 3.3 *±* 0.1 nm (Fig. S2*B*). However, the Rh value of Lipo-YcjN was 70 *±* 5 nm, which is much larger than both MBP and ΔYcjN (Fig. S2*C*, left panel). We also used negative stain TEM to analyze the size and morphology of Lipo-YcjN aggregates (Fig. S2*C*, right panel). The micrograph shows that Lipo-YcjN forms heterogeneously sized and irregularly shaped aggregates. Together, analyses of the DLS measurements, SEC profiles, and the TEM micrograph suggest that Lipo-YcjN aggregates in solution, possibly through hydrophobic lipid-lipid interactions.

In addition, a comparison of the YcjN SEC profiles reveals that the elution volumes of the major peaks of Lipo-YcjN appear to be sensitive to the presence of LMNG (Fig. 2*B*, light and dark blue stars). In contrast, the elution volumes of ΔYcjN remain relatively similar with and without LMNG (Fig. 2*B*, green traces). These data suggest that LMNG interacts with Lipo-YcjN aggregates, possibly solubilizing them into smaller Lipo-YcjN:LMNG micelle-like structures with slightly higher peak elution volumes (the difference between the major peaks indicated by star markers is 13 ml).

### Overall structure of ΔYcjN

To investigate the structural details of YcjN, we used X-ray crystallography to determine its three-dimensional structure. We chose the ΔYcjN protein for these crystallization studies because it does not aggregate in solution. We determined the first crystal structure of ΔYcjN at 1.95 Å resolution using the structure of an uncharacterized SBP from *Enterobacter cloacae* (Protein Data Bank (PDB) ID: 7V09) for molecular replacement. Data collection and structure refinement statistics are listed in Table 1. The crystal structure was solved with P1 symmetry, and the asymmetric unit contained two molecules. A comparison of the two molecules in the asymmetric unit shows that they are very similar (RMSD of 0.182 Å across all 401 pairs).

**Table 1:** Data collection and refinement statistics

PDB ID	apo YcjN (8VQK)

Data collection ^*a*^	
Wavelength (*Å*)	0.97946
Space group	P 1
Cell dimensions	
a,b,c (*Å*)	63.22 63.29 74.31
*α*, *β*, *γ* (^*◦*^)	110.0 91.1118.7
Unique reflections	61,761 (6203)
Multiplicity	3.9
Completeness (%)	91.19 (90.35)
Mean I/sigma(I)	4.9
CC half%	99.8 (29.3)
Refinement	
Resolution (*Å*)	34.04–1.95
R-work/R-free	0.22/0.26
Number of nonhydrogen atoms	6501
Macromolecules	6139
Ligands	43
Solvent	319
Protein residues	803
RMS(bonds)	0.007
RMS(angles)	0.81
Ramachandran	
Favored (%)	98.25
Allowed (%)	1.63
Outliers (%)	0.13
Rotamer outliers (%)	2.23
Clashscore	5.91
Average B-factor	39.89

Numbers in parentheses correspond to the highest resolution shell.

**Figure 3:**
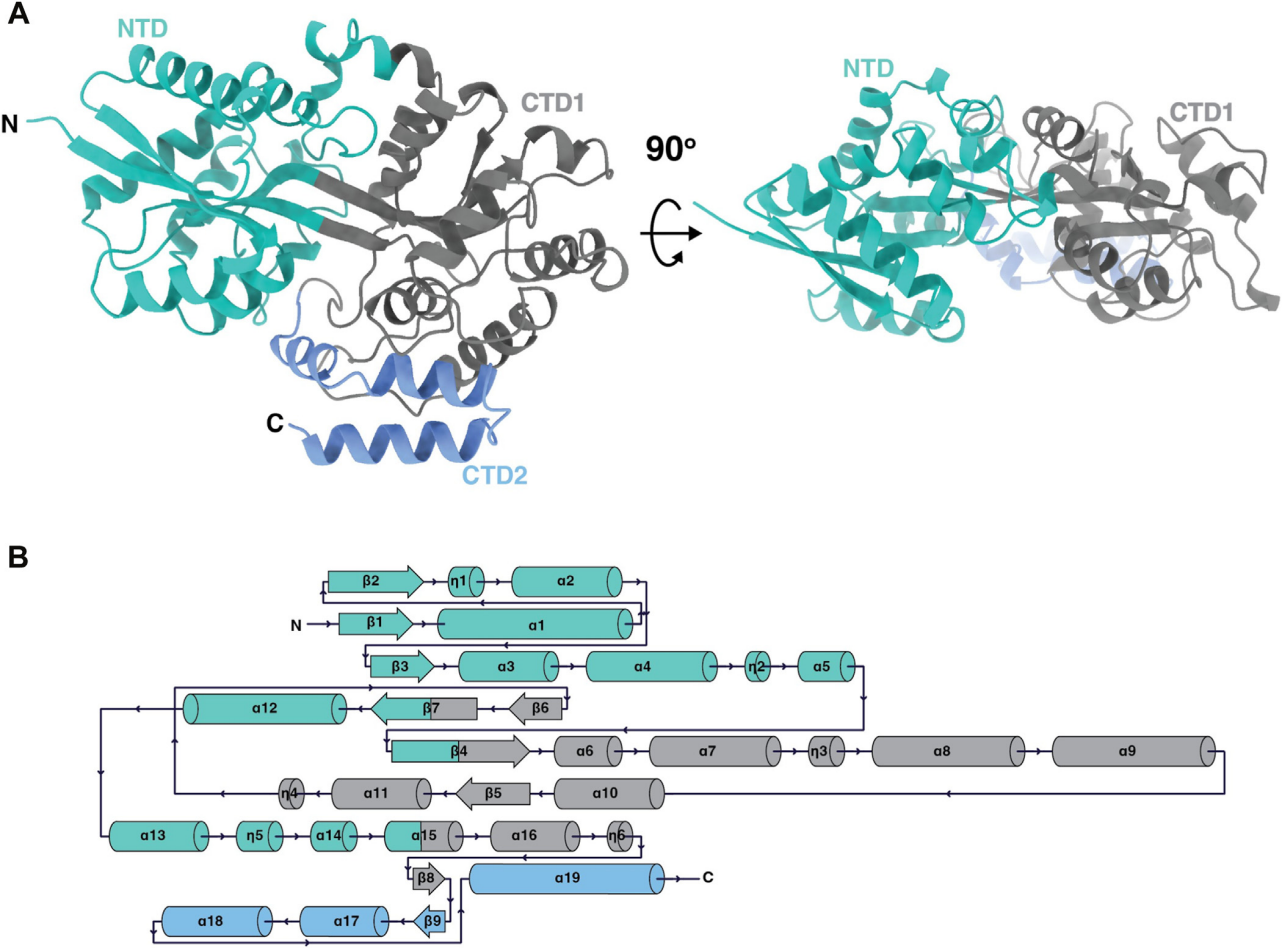
**Overall structure of *Escherichia coli* ΔYcjN**. *A*, ribbon representation of the ΔYcjN structure. The same structure is shown with a 90^*◦*^ x-axis rotation. *B*, schematic of secondary structure. Subdomains NTD, CTD1, and CTD2 are colored *teal*, *gray*, and *light blue*, respectively. CTD, C-terminal domain; NTD, N-terminal domain.

The YcjN structure is composed of two *α*/*β* domains: the N-terminal domain (NTD) and the C-terminal domain (CTD) that can be divided into two subdomains (CTD1 and CTD2) (Fig. 3, shown in teal, gray, and light blue, respectively). Each domain comprises two nonconsecutive amino acid segments (Fig. 3*B*). The NTD possesses a three-stranded *β*-sheet flanked by 11 *α*-helices (excluding linker segments). The CTD is composed of two subdomains (CTD1, residues 145–298 and residues 359–383; CTD2, residues 384–430) and is composed of three *β*-strands and 13 *α*-helices. The NTD and CTD are connected by three segments, including *β*7, *β*4, and *α*15. An analysis of the binding pocket revealed that there is no discernible electron density corresponding to a bound ligand deep within the binding pocket. However, a smaller electron density was detected at the edge of the binding pocket near tyrosine 95. We assign this electron density to PEG since it was included as an additive in the crystallization experiment (Fig. S3*A*).

### Comparison of YcjN to other SBPs

**Figure 4:**
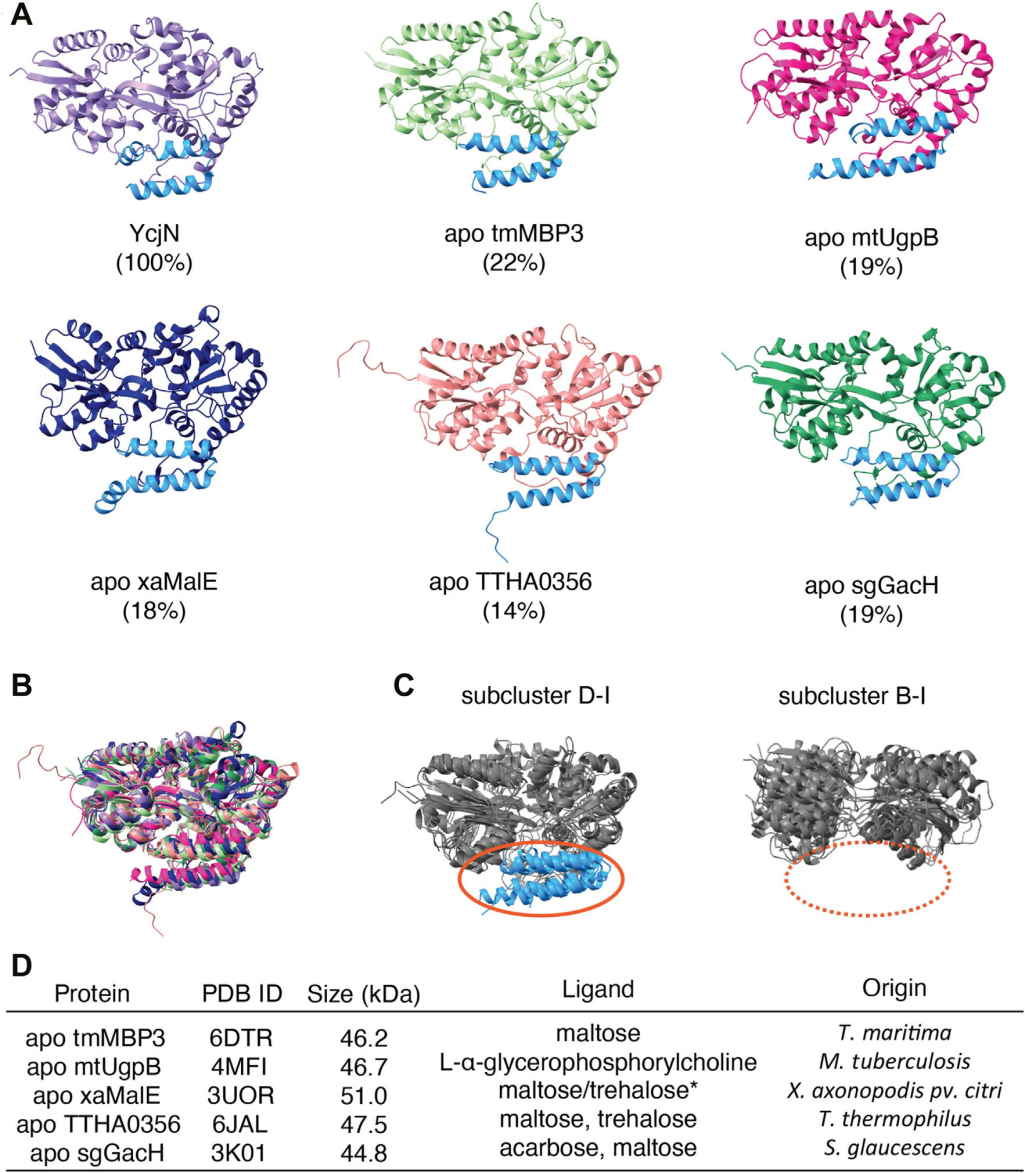
**Comparison of YcjN to other SBPs identified using the Dali Server**. *A*, structures and percent identities to YcjN. The CTD2 domain of each protein is colored *blue*. *B*, superposition of SBPs. Each protein color corresponds to that of the protein in *panel A*. *C*, SBP subcluster categorization of the aligned proteins. All CTD2 domains (*blue*) present in subcluster D-I (1ANF, 2ZYK, 4AQ4, and 2UVI) are absent in proteins within subcluster B-I (2FW0, 1GCG, 1URP, and 1GUB) that also bind carbohydrates. *D*, table of proteins, their size, ligand (∗putative), and species of origin. SBP, substrate binding protein; CTD, C-terminal domain.

A structural similarity search using the Dali server (14) revealed that YcjN is most closely related to putative carbohydrate-binding proteins. Top results from the search include various sugar binding proteins (Fig. 4), such as *Thermotoga maritima* tmMBP3 (15) [Z-score, 41.6; RMSD 2.4 Å for 393 residues, 22% identity], *Mycobacterium tuberculosis* mtUgpB (16) [Z-score, 38.7; RMSD 2.3 Å for 403 residues, 19% identity], *Xanthomonas. axonopodis pv. citri* xaMalE (17) [Z-score, 38.6; RMSD 2.6 Å for 397 residues, 18% identity], *Thermus thermophilus* TTHA0356 (18) [Z-score, 38.5; RMSD 2.5 Å for 416 residues, 14% identity], and *Streptomyces glaucescens* sgGacH (19) [Z-score, 38.4; RMSD 2.5 Å for 391 residues, 19% identity]. Notably, all of these proteins contain a distinct CTD2 domain (Fig. 4*A*, blue helices) and have molecular weights greater than 40 kDa (Fig. 4*D*). These characteristics are common among proteins belonging to subcluster D-I, which includes MBP (20, 21). SBPs that bind carbohydrates can also be members of subcluster B-I (11). However, these proteins lack the CTD2 domain, making them structurally distinct from subcluster D-I proteins (Fig. 4*C*). Due to YcjN’s molecular weight and the presence of the CTD2 domain, we group YcjN into subcluster D-I.

Top hits from the Dali search also have a low percent identity to YcjN (*<*25%) (Fig. 4*A*) and the volumes of their binding pockets vary (551 Å (3) to 1432 Å^3^, determined using CASTp 3.0 with a probe sphere 1.4 Å) (Fig. S3*B*). CASTp 3.0 determines binding pocket volume by identifying surface atoms that line the binding pocket and adding a negative volume imprint, which is shown as a surface representation. Furthermore, a sequence comparison did not reveal any strict identity of amino acid residues within the binding pockets for these proteins (Fig. S4). Despite variations in binding pocket volumes and low percent identities, three of the five proteins structurally similar to YcjN bind carbohydrates, including maltose, while another protein binds glycerophosphorylcholine (Fig. 4*D*). Therefore, it is plausible that YcjN could also bind similar ligands. However, experimental validation is necessary to identify YcjN’s ligand.

### NanoDSF screening to identify YcjN ligands

Unlike some SBPs, ΔYcjN did not crystallize bound to a ligand deep within its binding pocket. Therefore, to determine the ligand binding profile of ΔYcjN, we used thermal shift assay using nano-differential scanning fluorimetry (nanoDSF). This method uses tryptophan/tyrosine fluorescence at 330 nm and 350 nm to monitor protein thermal unfolding. These profiles can then be used to determine the protein’s denaturation midpoint (Tm), which reflects the stability of the protein or protein-ligand complex. In our experiments, the protein’s Tm was measured in the presence and absence of potential ligands and the ΔTm was calculated. The molecule was then classified as a ligand if the calculated ΔTm was greater than 2 °C.

**Figure 5:**
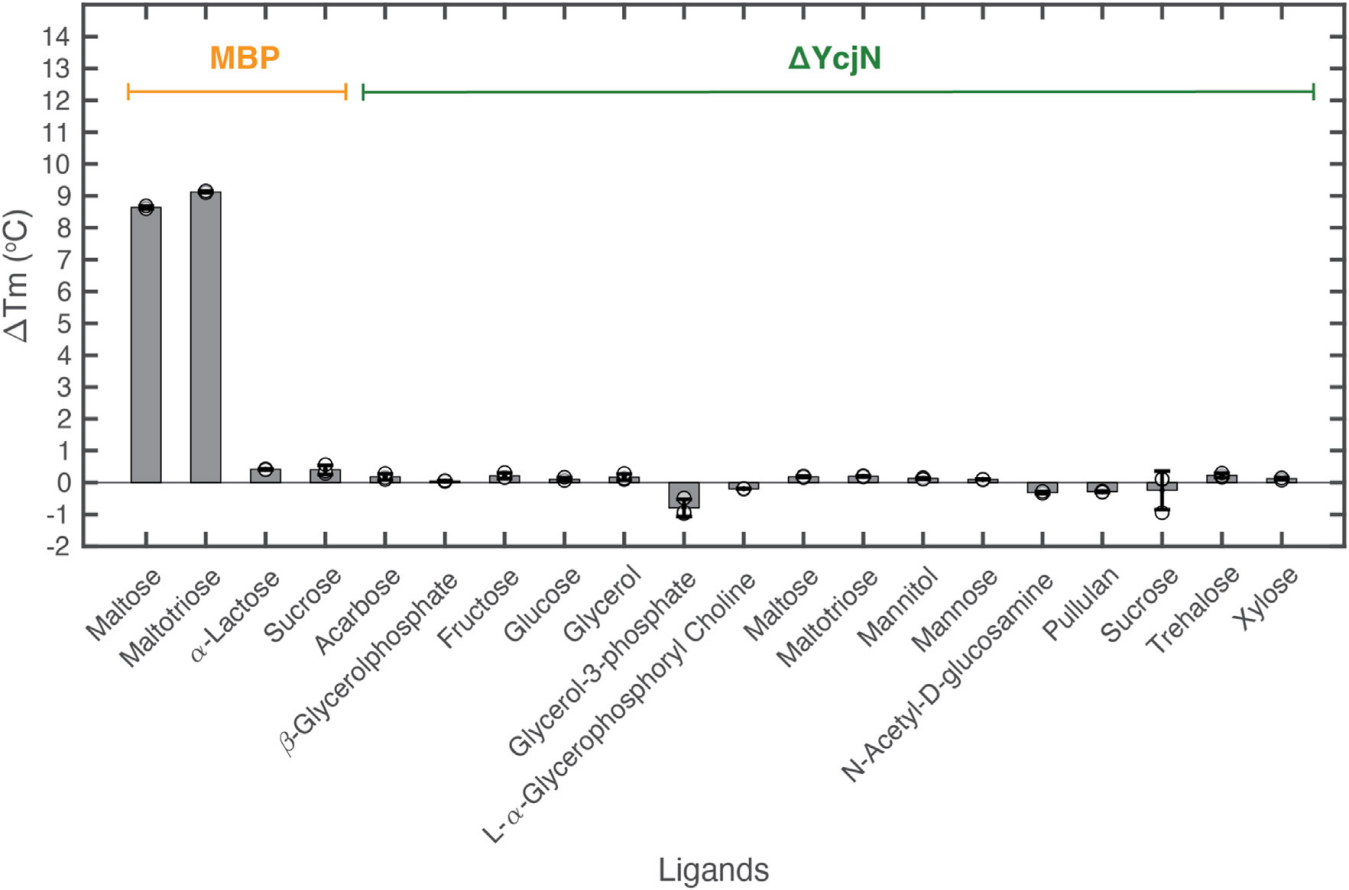
**NanoDSF thermal shift assay for MBP and ΔYcjN**. Bar graph displaying ΔTm for MBP and YcjN with potential ligands. All measurements were performed using final protein concentrations of 1 mg/ml (24 μM MBP and 22 μM ΔYcjN) and ligand concentrations of 5000 μM. Unfilled *circles* represent individual data points. The height of the bar represents the mean of the data set, and the error bars represent the standard deviation. Measurements were calculated using three technical replicates. MBP, maltose binding protein; nanoDSF, nano-differential scanning fluorimetry.

Since YcjN is part of a gene cluster encoding 12 proteins involved in carbohydrate metabolism, we included a range of commercially available monosaccharides and polysaccharides in our screen (Fig. 5). Considering the structural similarity of ΔYcjN to tmMBP3 and mtUgpB, we also included maltose and glycerophosphorylcholine in our screen. Additionally, the screen included kojibiose, a substrate for YcjT, which is an experimentally confirmed kojibiose phosphorylase with its gene located in the same operon as YcjN (12). As a positive and negative control, we calculated the ΔTm of MBP in the presence of two known ligands (maltose and maltotriose) and two nonligands (lactose and sucrose), respectively (22). As expected, the ΔTm of MBP increased by 8.6 °C and 9.1 °C in the presence of maltose and maltotriose, respectively, while in the presence of nonligands, lactose and sucrose, the ΔTm increased by less than 0.5 °C. Next, we monitored the thermal stability of ΔYcjN in the presence and absence of various potential ligands. These ligands did not produce a detectable thermostabilizing effect on ΔYcjN, with the ΔTm remaining below 0.5 °C for all screened ligands. Together, these data suggest that ΔYcjN’s ligands may not have been part of our screen or had no detectable thermostabilizing effects on ΔYcjN. As a result, the identity of YcjN’s ligands remains unknown.

### Bacteria and Archaea contain nonlipidated and lipidated YcjN-like proteins

**Figure 6:**
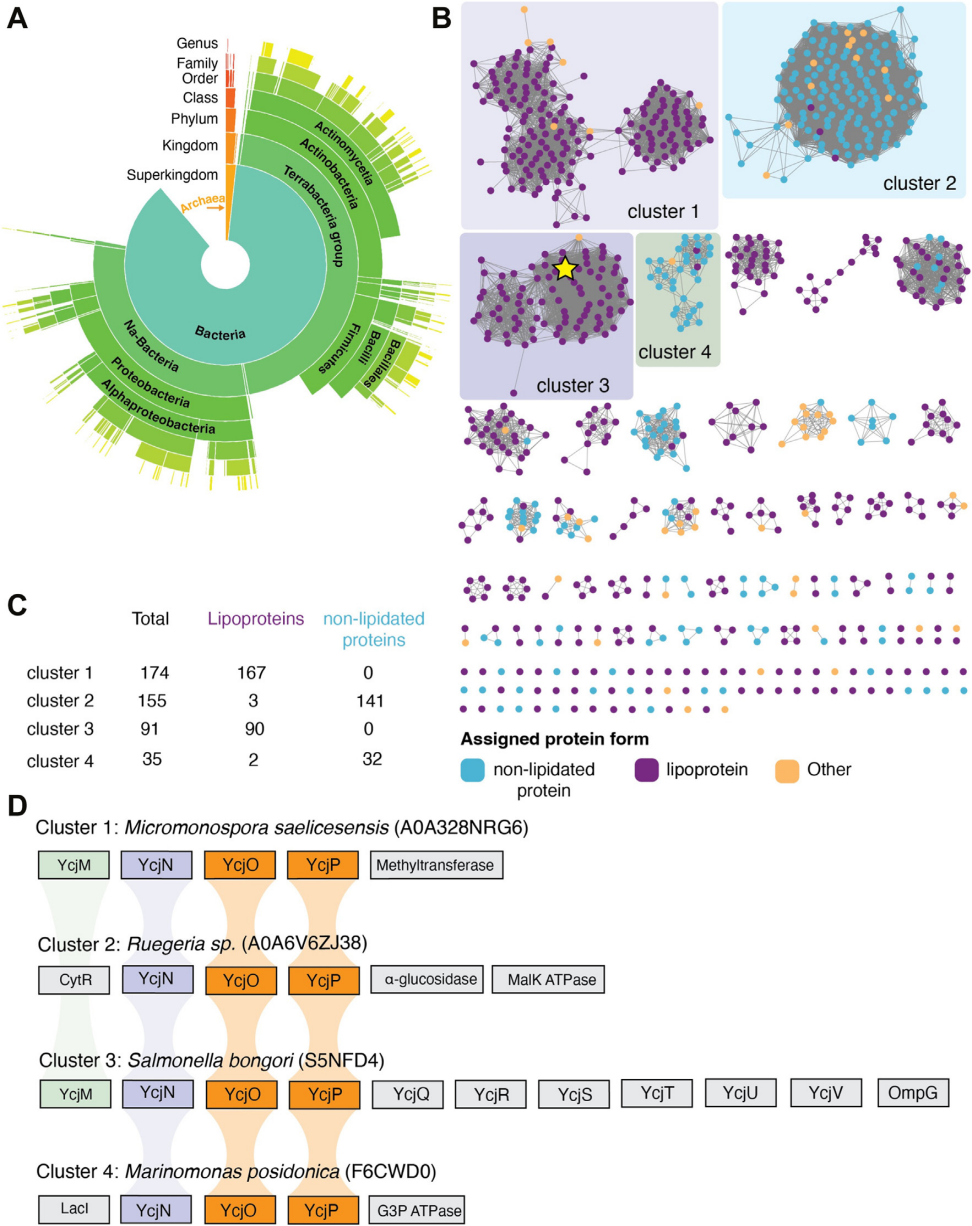
**Bioinformatic analysis of YcjN**. *A*, sunburst diagram of the taxonomic distribution of YcjN homologs. *B*, the SSN diagram. *C*, total number of proteins labeled as lipoproteins and nonlipidated proteins in clusters 1 to 4. *D*, representative diagrams of the putative proteins within clusters 1 to 4 that are frequently encoded near *YcjN* as determined using the EFI-GNT tool. Putative YcjM, YcjN, YcjO/YcjP proteins are colored in *green*, *purple*, and *orange*, respectively. SSN, sequence similarity network.

To determine whether lipidated YcjN-like proteins are exclusively present in Enterobacteriaceae such as *E. coli*, we investigated the taxonomic distribution and putative lipidation state of these proteins using bioinformatic tools. Specifically, to identify YcjN homologs, we used the Enzyme Function Initiative (EFI)-Enzyme Similarity Tool program to create a sequence similarity network (SSN) for YcjN and its closest homologs in the UniRef90 database. In the SSN, each protein is depicted as a node (circles), and if they exhibit pairwise protein sequence similarity, the nodes are connected by edges (lines). To identify putative lipidated and nonlipidated forms of YcjN homologs, we used SignalP 6.0 (https://services.healthtech.dtu.dk/services/SignalP-6.0/). This algorithm predicts which enzymes are likely to be involved in the posttranslational modification of immature proteins based on the protein’s amino acid sequence. Nodes in the SSN were manually annotated and colored as lipoproteins in purple, nonlipidated proteins in light blue, and other in orange, if SignalP 6.0 predicted that they were processed by SPase I, SPase II, or classified as other, respectively (Table S1). This analysis also produced a sunburst diagram with the taxonomic distribution of YcjN-like proteins (Fig. 6*A*). An inspection of this diagram suggests that YcjN homologs are primarily found in both Archaea and Bacteria. In addition, inspection of the SSN reveals that YcjN could be in a lipidated or nonlipidated form, suggesting that lipidation may not be essential for YcjN’s function. Furthermore, these proteins separated into several distinct clusters, with either lipidated or nonlipidated YcjN protein forms dominating each cluster (Fig. 6, *B* and *C*). For example, YcjN from *E. coli* (Fig. 6*B*, cluster 3, star marker) is predicted to share a high sequence similarity to other putative YcjN lipoproteins.

Next, we analyzed the genomic context of four clusters (1–4) to investigate the cooccurrence of genes near *YcjN*. This analysis showed that cluster 3 exhibited the most similar cooccurrence of genes to that of *E. coli*, with genes encoding proteins YcjM, YcjN, YcjO, YcjP, YcjQ, YcjR, YcjS, YcjT, YcjU, YcjV, and OmpG (Fig. 6*D*). This observation is not surprising since *E. coli* YcjN belongs to cluster 3 and shares the highest protein similarity to YcjN proteins within this group. Moreover, all four clusters contained putative genes encoding proteins YcjO and YcjP (Fig. 6*D*, orange), and two of the four clusters also included a gene encoding protein YcjM (Fig. 6*D*, green). These data suggest that the gene encoding YcjN often cooccurs with genes encoding the TMDs of an ABC transporter and, occasionally, next to a gene encoding a sugar phosphorylase. A closer inspection of cluster 3 reveals that it comprises various bacteria from the phylum *Proteobacteria* such as *Citrobacter farmeri*, *Shigella boydii*, and *E. cloacae*, as well as from the *Firmicutes* phylum, including *Enterococcus pseudoavium*, *Listeria fleischmannii*, and *Bacillus* sp. Many of these bacteria have been identified in food, soil, and humans/animals. Notably, none of the proteins within this cluster have been experimentally characterized, and their ligands remain unknown. Together, these data suggest that YcjN may be involved in carbohydrate import, with the closest homologs of YcjN found in several bacteria that inhabit animals or soil.

## Discussion

Nutrient acquisition is a fundamental process essential for bacterial growth and survival. To scavenge nutrients, bacteria use several mechanisms, including the active transport of molecules using SBPs and their cognate ABC transporters. *E. coli* possesses several SBPs with distinct ligand binding profiles, allowing the bacterium to adapt to environments with varying nutrient compositions. To gain structural insight into the substrate binding protein, YcjN, we recombinantly overexpressed, purified, and characterized lipidated and nonlipidated YcjN from *E. coli*, named Lipo-YcjN and ΔYcjN, respectively. Specifically, we report the first crystal structure of ΔYcjN determined at 1.95 Å resolution. In the crystal structure, the binding pocket is empty except for a PEG molecule bound to the outer edge of the binding pocket. Most likely, this PEG molecule was introduced during the crystallization experiment. A structural similarity search showed that ΔYcjN closely resembles sugar-binding proteins and an analysis of its structure revealed that it can be classified into subcluster D-I, which comprises SBPs of molecular weights greater than 40 kDa and a distinct CTD2 domain. Notably, this group includes MBP.

Unlike some SBPs, ΔYcjN did not crystallize bound to a ligand deep within its binding pocket. Moreover, CASTp3.0 analysis of structurally similar proteins revealed no discernable trend between ligand binding pocket volume and ligand. Therefore, to determine the ligand binding profile of ΔYcjN, we used a nanoDSF thermal shift ligand binding assay. Despite our efforts, our screen did not reveal any ligand that produced a thermostabilizing effect greater than 2 °C. Specifically, no binding was detected for known ligands of structurally similar SBPs, such as maltose and glycerophosphorylcholine. We also included several other sugars in our assay, which were chosen based on experimentally characterized substrates for proteins within the operon. For example, YcjT is a kojibiose phosphorylase, YcjU is a β-D-glucose 1-phosphate β-phosphoglucomutase, YcjM is an α-D-glucosyl-2-glycerate phosphorylase, YcjQ is a D-glucoside dehydrogenase, YcjR is a 3-keto-D-glucoside isomerase, and YcjS is a 3-keto-D-glucoside dehydrogenase (23). We chose to include kojibiose and glucose in the screen since they were commercially available. However, we did not test the binding of glucose analogs or glucoside, and therefore cannot rule out these molecules as potential ligands. Importantly, the lack of a thermostabilizing effect does not indicate the absence of ligand binding. Because our assay does not measure ligand binding directly, it is possible that ΔYcjN binds these ligands but does not produce a measurable thermostabilizing effect. However, we believe that it is more likely that YcjN’s endogenous ligand was absent from our screen because YcjN shares structural similarity to MBP which, in our assay, clearly showed a thermostabilizing effect in the presence of known ligands maltose and maltotriose. Given that our bioinformatic analysis suggests that YcjN-like proteins are found in food, animals, or soil dwelling bacteria with its gene cooccurring proximal to a sugar phosphorylase, we speculate that its ligand could be a plant-derived carbohydrate, glycolipid, or metabolite. Consequently, future efforts should consider using an alternative screening method or broadening the classes of ligands in the screen.

Our results also show that Lipo-YcjN aggregates in solution, whereas its nonlipidated form does not. Similar observations have been made for other lipoproteins. For example, the SEC elution profiles of lipidated *N. meningitidis* MetQ and lipidated *Borrelia burgdorferi* OspA reveal lower peak elutions compared to their nonlipidated counterparts (5, 24). Moreover, studies on the lipoprotein *N. meningitidis* fHbp using SEC with multiangle light scattering revealed a molecular weight of 660 kDa, which is much higher than the theoretical weight of its monomer (28 kDa) (25). Together, these findings suggest that lipid-mediated aggregation may be an intrinsic property of bacterial lipoproteins. While the biological relevance of lipoprotein aggregation within the cell may be limited since lipoproteins anchor to cellular membranes in bacteria, understanding lipoprotein properties in solution may be important for *in vitro* studies. For example, *B. burgdorferi* OspA and *N. meningitidis* fHbp are two recombinantly expressed lipoprotein antigens included in vaccine formulations. Importantly, their lipid moieties play a key role in inducing a robust immune response (24, 26). Therefore, a comprehensive understanding of lipoprotein aggregation in solution may be important for the effective separation of lipidated and nonlipidated protein forms using chromatography techniques, a key step in isolating lipidated antigens for vaccine development.

Our study also details protocols for purifying Lipo-YcjN both with and without LMNG.

Previous studies have used a variety of detergents, including Triton X-100 and n-dodecyl-β-D-maltoside, to purify histidine-tagged lipoproteins *via* affinity chromatography (5, 27). However, here we demonstrate that Lipo-YcjN can be purified in the absence of detergent. This result suggests that detergents are not an essential component for the purification of all lipoproteins. In addition, analysis of the SEC profiles for Lipo-YcjN in the presence and absence of LMNG revealed a higher peak elution volume compared to that of its nonlipidated form. These data suggest that LMNG may solubilize lipoprotein aggregates, possibly into smaller lipoprotein-LMNG mixed micelles. However, the interactions between detergents and lipoproteins remain poorly understood and require further investigation. Another key finding of our study is that recombinantly produced YcjN is predominantly observed in its diacylated form rather than in its triacylated form. Specifically, mass analysis of the major Lipo-YcjN peak fractions purified with and without detergent failed to detect any high-intensity masses corresponding to a triacylated Lipo-YcjN form. This finding is surprising since the prevailing view in the field is that bacteria that possess an Lnt homolog, such as *E. coli*, are expected to produce triacylated lipoproteins. In contrast, Gram-positive bacteria that lack Lnt homologs are expected to produce diacylated lipoproteins. This view was supported by the characterization of *E. coli's* most abundant lipoprotein, LPP, which was previously shown to be triacylated (28). However, recent studies in Gram-positive bacteria have begun to challenge this general assumption. For example, studies have identified triacylated lipoproteins in Gram-positive bacteria that lack identifiable Lnt homologs (29). However, an analysis of whether all natively produced lipoproteins are diacylated and/or triacylated in Gram-negative bacteria, such as *E. coli*, has not been carried out.

In contrast to studies on natively produced bacterial lipoproteins, several studies are available on recombinantly overexpressed lipoproteins produced in an *E. coli* expression system. These studies have found that expression conditions, such as media composition and pH, can modulate the production of diacylated and triacylated lipoprotein forms, suggesting overexpression conditions can influence the production of diacylated and triacylated lipoprotein forms (30). However, we previously overexpressed *N. meningitidis* MetQ, using the same expression protocol, and we only detected triacylated Lipo-NmMetQ. Together, these studies suggest that environmental conditions, and possibly the lipoprotein structure or amino acid sequence, could influence the production of diacylated *versus* triacylated lipoprotein forms. However, the molecular determinants for producing exclusively diacylated or triacylated lipoproteins have not been fully elucidated. Our results, in light of previous studies, raise several questions about the biosynthesis and processing of lipoproteins: Could the expression of specific proteins influence environmental conditions, thereby favoring the formation of diacylated lipoproteins? Alternatively, could the N-terminal amino acids, such as the signal peptide, trigger an early exit from the lipoprotein maturation pathway? Are diacylated lipoproteins an uncharacterized form in *E. coli*, or are they just artifacts of recombinant overexpression methods? Given that these possibilities are not mutually exclusive, two or more factors could help explain the preference for endogenous and recombinant production of diacylated over triacylated lipoproteins in *E. coli*. In addition, our results highlight the need to characterize the lipidation state of natively produced bacterial lipoproteins, such as YcjN, in Gram-negative bacteria.

In summary, this study contributes to our overall understanding of Lipo-YcjN, providing a foundation for future research on bacterial lipoproteins. These findings are particularly relevant for future studies that aim to characterize bacterial lipoproteins in solution and to help advance methods for the production of recombinant, lipidated proteins for vaccine formulations.

## Experimental procedures

### Cloning, expression, and purification of YcjN and MBP proteins

The amino acid sequence of YcjN was obtained from *E. coli* K-12 (gene ID 945696; UniProt ID P76042). To produce YcjN constructs, the DNA sequence encoding YcjN was inserted into a pET21b(+) ampicillin-resistant vector between NdeI and XhoI sites and under the control of a T7 promoter (GenScript Biotech). Two constructs were created: SP-YcjN-10H, 6H-ΔYcjN (residues 1–21 removed). To aid in purification, nucleotide sequences encoding either decahistidine (10H) or hexahistidine (6H) were added to the N or C terminus of the *YcjN* genes. Similarly, the amino acid sequence of MBP was obtained from *E. coli* K-12 (gene ID 948538; UniProt ID P0AEX9). In this construct, residues 1 to 26 encoding the SP were replaced with a 6H. All proteins were expressed in *E. coli* BL21 (DE3) gold cells (Agilent Technologies) using ZYM-5052 autoinduction media (31) containing 100 mg/L ampicillin at 37 °C for 30 h. Cells were harvested by centrifugation at 4785*g* (JLA 8.1; 5000 rpm; Beckman Coulter) for 15 min, and the cell paste was flash frozen in liquid nitrogen for storage at −80 °C.

To purify YcjN and MBP proteins, *∼*10 g of cell paste was thawed at room temperature, and then placed in solution of 100 ml of 25 mM Tris–HCl pH 7.5 and 100 mM NaCl, 40 mg of lysozyme, 4 mg of DNase, and one cOmplete protease inhibitor cocktail tablet (Sigma-Aldrich). The suspended cells were disrupted using a Microfluidizer (Microfluidics), and cell debris was removed by centrifugation at 7823*g* (Ti45; 10,000 rpm; Beckman Coulter) for 30 min. Imidazole was then added to the lysate to remove nonspecific protein binders during affinity purification (25 mM imidazole for 6H constructs; 70 mM imidazole for 10H constructs). Proteins were purified using a 5 ml HisTrap HP column (Cytiva) preequilibrated with 25 mM Tris–HCl pH 7.5 and 100 mM NaCl and eluted with a solution of 25 mM Tris–HCl pH 7.5, 100 mM NaCl, and 300 mM imidazole. The eluted proteins were then loaded onto a Hiload 16/600 Superdex 200 pg (Cytiva) preequilibrated with 25 mM Tris–HCl pH 7.5 and 100 mM NaCl. YcjN proteins purified in the presence of LMNG were obtained using the same protocol with the following modifications: After cell disruption, LMNG was added to the lysate at a final concentration of 1% and incubated at 4 °C for 3 h. Cell debris was removed by centrifugation at 94,834*g* (Ti45; 35,000 rpm; Beckman Coulter). For affinity and size exclusion chromatography, the same protocols were used with buffers containing LMNG added to a final concentration of 0.01%. For all proteins, the peak fractions were combined, frozen in liquid nitrogen, and stored at −80 °C until thawed. Mass analysis of intact YcjN proteins was performed as previously described (5). The mass spectrum graph was imported into Adobe Illustrator (CC 2024), where text and font size were modified to increase readability. Graphs were generated in Matlab (version R2022b; https://www.mathworks.com/products/matlab.html).

### Negative stain TEM and dynamic light scattering

Lipo-YcjN protein was diluted to a final concentration of 0.01 mg/ml and adsorbed onto glow-discharged Formvar/Carbon 200 mesh, copper grids (Ted Pella, Inc) for 20 s, and then blotted using dry filter paper. The sample was stained by placing the grid against the surface of a 10 μL drop of 1% uranyl acetate (Electron Microscopy Sciences) on parafilm four times, blotting in between each staining step, and then left on filter paper to fully dry. Micrographs were obtained with 250,00× magnification using Morgagni 100 kV TEM equipped with an Orius Camera (FEI).

Dynamic light scattering measurements of Lipo-YcjN were performed using SEC peak fractions using the default Measure Size option on a DynaPro NanoStar II (Wyatt Technology Corporation). A total of five measurements per sample were acquired. Hydrodynamic radii values were then exported, and Matlab was used to calculate the mean and standard deviation of the measurements. This software was also used to plot the representative DLS profile.

### Crystallization, data collection, structural determination, and structural analysis of ΔYcjN

The pooled factions of ΔYcjN were concentrated to 175 mg/ml for crystallization trials. The ΔYcjN protein was crystallized at 16 °C by the sitting-drop vapor diffusion method using a precipitant solution from the Basic Chemical Space Screen (Molecular Dimensions Inc) consisting of 0.01 M CdCl_2_, 0.2 M NH_4_SO_4_, 0.1 M Hepes pH 7.5, and 25% w/v mix consisting of PEG 3350, PEG 4000, PEG 2000, and PEG 5000 poly(ethylene glycol) methyl ether 5000. The crystals were cryocooled in liquid nitrogen after dipping them into a cryoprotectant solution composed by the precipitant solution supplemented with 25% PEG 400. The X-ray diffraction data were collected on SSRL beamline 12-1 at the Stanford Synchrotron Radiation Lightsource (SSRL) at the SLAC National Accelerator Laboratory, processed with the XDS package (https://xds.mr.mpg.de/) (32), and scaled and merged with SCALA (33). The ΔYcjN protein crystal belonged to the P1 space group and the structure was determined by molecular replacement with PHASER (https://phenix-online.org/documentation/reference/phaser_mr.html) (34) using the coordinates of *E. cloacae* (PDB ID: 7V09, 75% identity) an uncharacterized protein as the search model. The model was manually adjusted using Coot (https://www2.mrc-lmb.cam.ac.uk/personal/pemsley/coot/) (35) and refined using Phenix.refine (36). The atomic coordinates and structure factors have been deposited in the RCSB Protein Data Bank (8VQK). Figures were created by using PDBsum (37) to generate secondary structural schematics and ESPript 3.0 (https://espript.ibcp.fr/ESPript/ESPript/index.php) (38) to obtain secondary structure labels. These elements were manually integrated using Adobe Illustrator (https://www.adobe.com/home) to produce the final figure.

The visualization of the protein binding pockets and the identification of the residues lining the pockets were performed by the CASTp 3.0 server, using default values (39). The CASTp 3.0 server used negative volume, which is the space encompassed by the atoms that form geometric and topical features (ex. substrate binding pocket). To calculate the volume and area metrics, CASTp 3.0 used both the solvent accessible surface model and the molecular surface model. Multiple sequence alignment was performed using the align functionality in UniProt and ESPript 3.0 was used to visualize the alignment in the Black and White scheme. In the final sequence alignment, the residues lining the pockets were manually colored in red using Adobe Illustrator. Visualization of YcjN electron density maps was performed using ChimeraX (40) or the Moorhen website (https://moorhen.org). The protein similarity search was performed using the DALI server against the PDB90 database.14 The top protein hit, 7V09 with 75% identity, was excluded from the structural comparison since it was used as the model for molecular replacement.

### NanoDSF

To remove potential endogenously bound ligands, we purified ΔYcjN and MBP and subjected both proteins to dialysis, using a protocol similar to that previously described (41). The protein ΔYcjN was chosen for these studies because the thermal profile of lipid-modified YcjN may be a convolution of aggregate disassembly and protein unfolding, which could pose challenges in data analysis. Briefly, after purification, dialysis was performed using a 12 ml Slide-A-Lyzer Dialysis Cassette with a molecular weight cut-off of 3.5 K (Thermo Fisher Scientific). The dialysis was conducted at 4 °C for a total duration of 48 h. During this period, proteins were dialyzed against 2 L of a solution containing 25 mM Tris–HCl pH 7.5 and 100 mM NaCl, and the dialysis solution was replaced once after 24 h.

NanoDSF was performed on a Prometheus Panta (NanoTemper Technologies Inc). The 330/350 nm fluorescence ratio was recorded between 25 °C and 70 °C at 1 °C/min. Measurements were made using Prometheus nanoDSF Grade standard capillaries using excitation power of 20%. All measurements were performed using protein stock solutions of 2 mg/ml (48 μM MBP and 44 μM ΔYcjN) and 10 mM ligand stock solutions in 25 mM Tris–HCl pH 7.5 and 100 mM NaCl. For ligand binding experiments, protein stock solutions were mixed with an equal volume of ligand stock solution at a 1:1 v/v ratio to give a final solution of 1 mg/ml (24 μM MBP and 22 μM ΔYcjN) with 5 mM ligand in 25 mM Tris–HCl pH 7.5, 100 mM NaCl. The ligand free sample was prepared using 1:1 v/v ratio with buffer to give a final solution of 1 mg/ml protein concentration. All sugars, glycerol, β-glycerophosphate disodium salt hydrate, sn-glycerol-3-phosphate bis (cyclohexylammonium) salt, and sn-glycero-3-phosphocholine 1:1 cadmium chloride adduct were all purchased from Sigma-Aldrich, with the exception of acarbose, which was purchased from Thermo Fisher Scientific.

### Bioinformatics and data analysis

The sequences were obtained using EFI-Enzyme Similarity Tool (42, 43) using the BLAST retrieval option (Option A) with the Uniref90 database and the FASTA sequence of the YcjN protein, which was obtained from UniProt (44). Taxonomy categories included the superkingdoms of Bacteria, Eukaryota, Viruses, and Archaea. The metagenome species and the phylum that fell in the unclassified fungi category were excluded. The final SSN was created using an alignment score threshold of 150 and a sequence restriction minimum of 400. The SSN contained 909 nodes with more than 40% sequence similarity to the YcjN protein. SignalP 6.0 was then used to analyze the FASTA sequences of the SSN (45). SignalP 6.0 produced a summary of 896 predicted sequences, which included their respective type of signal peptide. Proteins predicted to be processed with SPase I or SPase II, and other were annotated as non-lipidated proteins, lipoproteins, or other, respectively. Protein sequences that failed the SignalP 6.0 analysis were removed from the SSN. The final SSN contained 896 protein sequences (Data S1) and was visualized using the organic layout on Cytoscape (https://cytoscape.org). The taxonomy distribution sunburst was imported into Adobe Illustrator, where text and font size were modified to increase readability. EFI-GNN tools were used to analyze the genome context of YcjN-like proteins. The output of the EFI-GNN analysis was used to manually create protein diagrams in Adobe Illustrator.

## Data availability

The entire data are provided in the manuscript. Structural data of ΔYcjN have been deposited in the RCSB Protein Data Bank with the accession code 8VQK.

## Supporting information

This article contains supporting information.

## Conflict of interest

The authors declare that they have no conflicts of interest with the contents of this article.
