# Supplementary material for: Expression, purification, and characterization of diacylated Lipo-YcjN from Escherichia coli
Source: J Biol Chem. 2024 Oct 1;300(11):107853. doi: 10.1016/j.jbc.2024.107853 (PMC11543891; doi:10.1016/j.jbc.2024.107853)
Supplement: Supporting Information [file mmc2.docx]

# Supporting Information

_
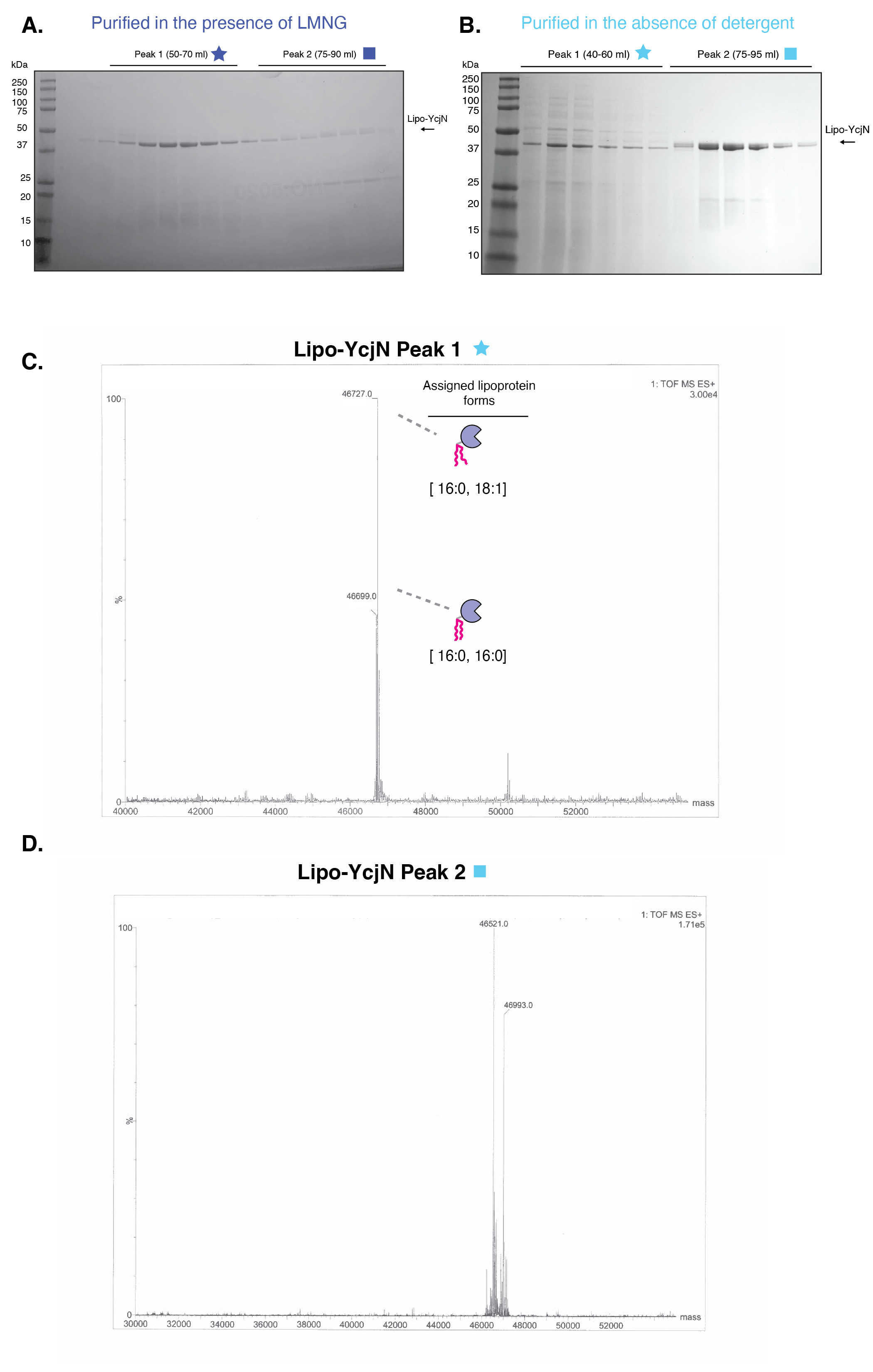
_

**Figure S1:** Analysis of SEC peak fractions using SDS-PAGE with ReadyBlue protein gel stain. A. Lipo-YcjN purified in the presence and B. absence of LMNG. C. Mass spectra of the first peak faction of Lipo-YcjN (elution volume of 49 mL, light blue star) and D. the second peak fraction of Lipo-YcjN (elution volume 82 mL, light blue square) purified in absence of LMNG.


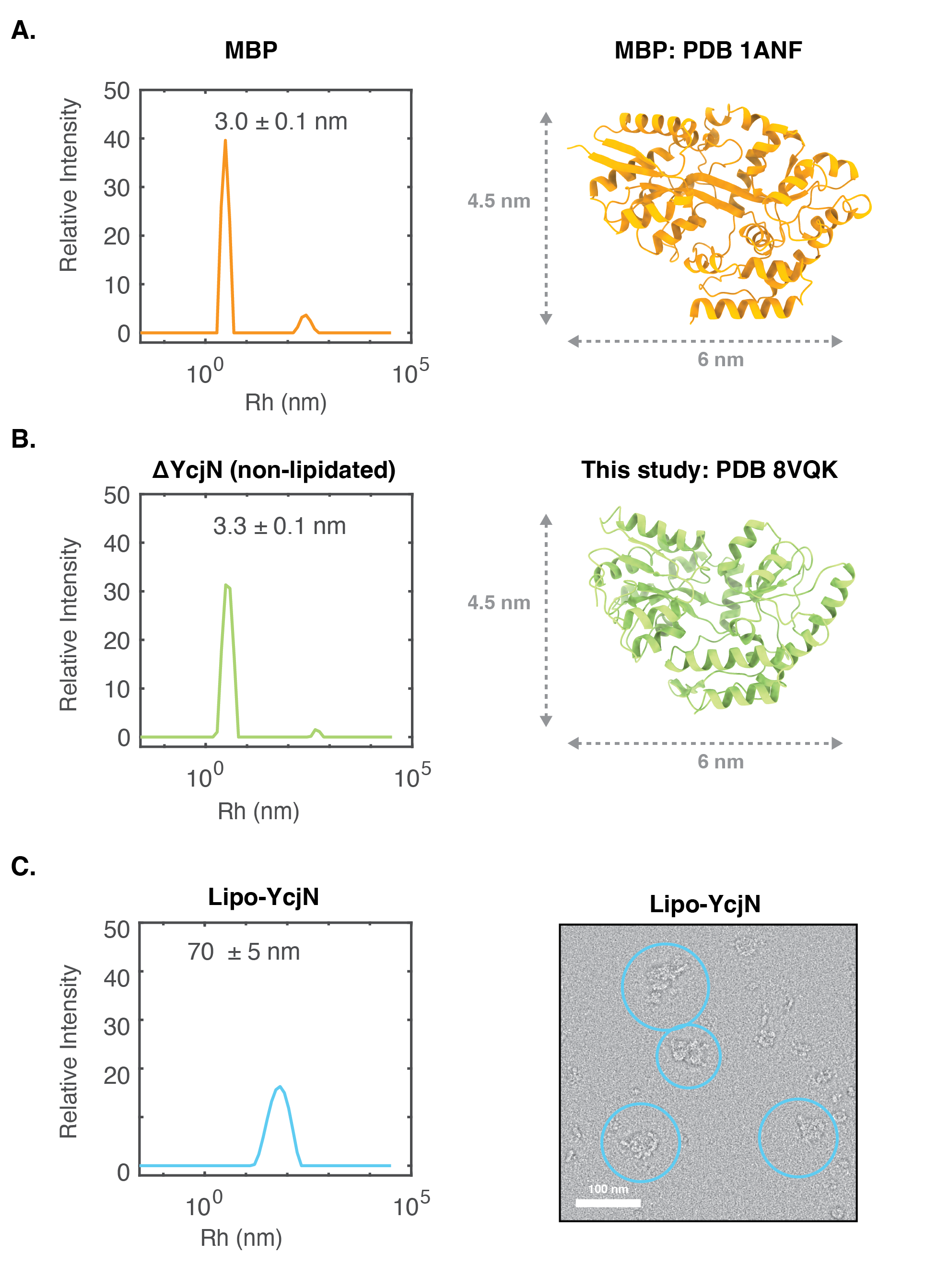


**Figure S2:** DLS and TEM analyses of YcjN and MBP proteins. A. DLS plot of MBP (left panel) and structure of MBP shown in ribbon representation (right panel). Approximate measurements of height and width are indicated by grey arrows. B. DLS plot of ∆YcjN purified in the absence of detergent (left panel) and structure of ∆YcjN shown in ribbon representation (right panel) and C. DLS plot of Lipo-YcjN purified in the absence of detergent and representative negative stain TEM micrograph. DLS analyses were carried out by acquiring n=5 individual measurements, values shown as mean*±* SD).


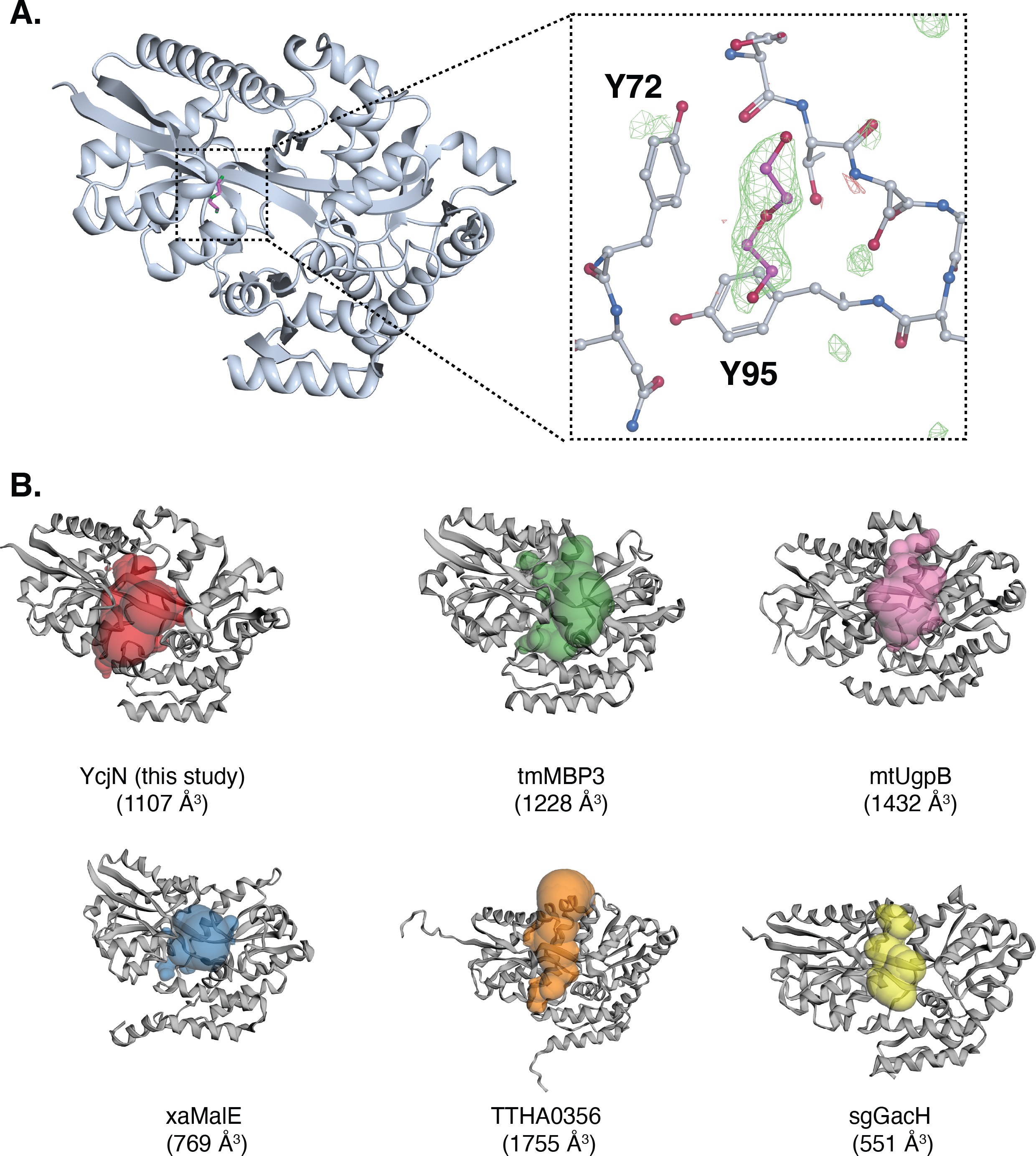


**Figure S3:** Ligand binding pockets of YcjN and YcjN homologs. A. Overall structure of YcjN (left panel) and an enlarged view of the binding pocket (right panel). In the enlarged view, the molecular model is depicted in stick representation. The Fo-Fc (2.6 RMSD) maps is shown in green/red meshes. PEG (magenta) was assigned to the electron density detected near the outer edge of the YcjN binding pocket near tyrosine 95 and tyrosine 72. No other unassigned electron densities were detected in the binding pocket. B. Ligand binding pockets of tmMBP3, mtUgpB, xaMalE, TTHA0356, and sgGacH proteins. All proteins are shown in gray ribbon representations and the negative volume imprints of their binding pockets are displayed in different colors, as determined by CASTp 3.0.


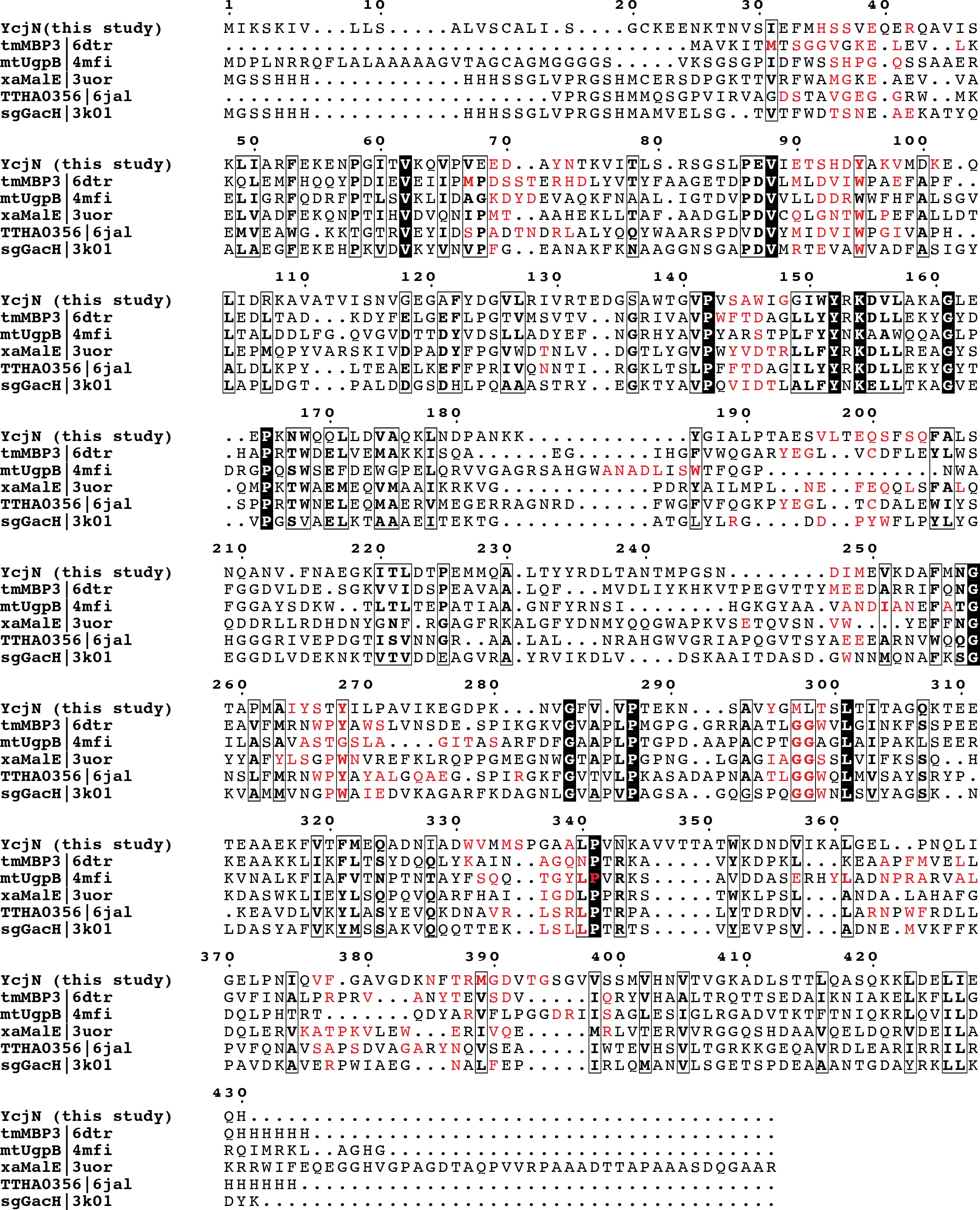


**Figure S4:** Sequence alignment of YcjN against top Dali hits. In the figure, white characters in a black box indicate strict identity, bolded characters indicate similarity within a group, a black frame indicates similarity across groups, and residues lining each protein’s binding pocket, as determined by CASTp 3.0, are colored in red.
